# Prevalence and Associated Risk Factors for Intestinal Parasitic Infections Among Schoolchildren at Mikara Primary School, Northwest Ethiopia

**DOI:** 10.1155/bmri/8017454

**Published:** 2026-02-25

**Authors:** Gebre Ayanaw Alula, Yideg Abinew

**Affiliations:** ^1^ Department of Biology, College of Natural and Computational Science, Debark University, Debark, Ethiopia, dku.edu.et; ^2^ Department of Nursing, College of Health Science, Debark University, Debark, Ethiopia, dku.edu.et

**Keywords:** intestinal parasitic infection, Mikara Primary School, prevalence, risk factor, schoolchildren

## Abstract

Intestinal parasitic infections (IPIs) are major public health problems with a wide global distribution and are associated with significant diseases and death, especially in preschool‐aged and school‐aged children. This study is aimed at assessing the prevalence of IPIs and at identifying related risk factors among schoolchildren enrolled at Mikara Primary School in Ethiopia. A school‐based cross‐sectional study was conducted from February to May 2024. A total of 363 schoolchildren participated in the study, selected through stratified random sampling. Data on risk factors for IPIs were collected using a structured questionnaire, and stool samples were obtained and analyzed by direct wet mounting and sedimentation methods. Logistic regression analysis was used to assess possible relationships between the dependent and independent variables. In this study, the overall prevalence of IPIs was 40.5% (147 out of 363 participants), and seven different species of intestinal parasites were identified. *Entamoeba histolytica/disar* was the most common species, with a prevalence of 10.7%, followed by *Giardia lamblia* (8.8%), *Ascaris lumbricoides* (8.5%), hookworm (7.2%), *Trichuris trichiura* (4.1%), and Taenia spp. (2.8%) and *Enterobius vermicularis* (1.7%). The probability of having an IPI was higher in men (adjusted odds ratio [AOR = 2.02]), children under 9 years of age (AOR = 3.22), individuals practicing open defecation (AOR = 3.2), those who relied on stream or river water as their main source of drinking water (AOR = 4.2), and participants who did not wash their hands before eating (AOR = 3.65). The occurrence of IPIs among study participants was notably high. Therefore, relevant stakeholders should design and implement evidence‐based intervention measures to effectively prevent and control IPIs throughout the study area, placing a particular focus on targeted health education and awareness campaigns for schoolchildren and their parents.

## 1. Introduction

Intestinal parasitic infections (IPIs) are among the most widespread public health concerns worldwide. They contribute significantly to disease and death, with preschool and school‐age children bearing a particularly heavy share of the burden [1, 2]. Globally, approximately 3.5 billion people are affected by intestinal parasites, with more than 450 million suffering from clinically important illnesses, leading to more than 200,000 deaths per year [3]. Intestinal helminths and protozoa are the main causative agents of these infections, especially in low‐income and middle‐income countries, where environmental and socioeconomic conditions strongly favor their spread [4]. IPIs occur much more frequently in tropical and subtropical areas, especially in sub‐Saharan Africa, because favorable climate, poor sanitation systems, unsafe drinking water, and particular sociocultural behaviors create conditions that greatly facilitate parasite transmission [5].

Children are disproportionately affected by IPIs due to their still‐developing immune systems, less effective hygiene practices, and relatively high nutritional requirements [6]. Infected individuals often present with growth delay, anemia, and protein–energy malnutrition, clinical outcomes that can ultimately lead to stunted linear growth, neurocognitive impairments, and reduced school performance [7, 8]. Across the world, millions of preschool and school‐age children live in areas with high transmission of soil‐transmitted helminth and protozoan infections, placing them at increased risk of long‐term negative health consequences and impeded physical, cognitive, and psychosocial development [9]. Key factors contributing to IPIs in children include inadequate sanitation facilities, overcrowded living and community environments, repeated contact with contaminated soil, poor hand washing practices, and reliance on microbiologically unsafe water supplies in both school and home settings [10].

In Ethiopia, IPIs represent a significant public health concern. It is estimated that around 81 million people live in areas where these infections are prevalent, including approximately 25.3 million school‐age children [11]. Epidemiological data show that IPIs are the second leading cause of outpatient illnesses nationwide [12]. The high rate of IPIs in Ethiopia is largely related to inadequate sanitation facilities, unsafe or contaminated drinking water, the common practice of open defecation, frequent walking barefoot, and limited access to health education [13]. Although IPIs have been widely reported in many parts of the country, several areas, including the one examined in this study, have not been adequately explored and currently have little or no epidemiological information available [13, 14]. The absence of current, context‐specific epidemiological and operational data restricts the capacity for evidence‐based planning and hinders the effective design, implementation, and evaluation of preventive and control interventions within school settings and surrounding communities.

In addition, although many IPIs remain asymptomatic, they can cause a wide range of clinical manifestations, such as chronic diarrhea, abdominal pain, nausea, vomiting, iron deficiency anemia, and protein–energy undernutrition [3]. These consequences significantly affect linear growth, cognitive development, and school performance in children [14]. A growing body of evidence shows that IPIs can worsen existing malnutrition and increase the host′s vulnerability to other simultaneous infections [15]. These observations highlight the vital need for early case identification, ongoing surveillance through routine screening, and the adoption of targeted, context‐appropriate interventions in areas where these infections are endemic [16].

Although the burden of IPIs and their harmful consequences for child health are well recognized, there is still a notable lack of comprehensive studies that simultaneously examine both their prevalence and related risk factors among school‐aged children in many parts of Ethiopia. Producing solid, context‐specific evidence on the extent of IPI and clarifying local determinants of infection is vital to designing effective and locally appropriate intervention strategies to mitigate this burden. In this context, the current study was carried out to estimate the prevalence of IPIs and to identify associated risk factors among schoolchildren enrolled at Mikara Primary School in northwest Ethiopia. The results are expected to provide practical evidence to local health officials, school leaders, and policymakers, thus informing the development and implementation of targeted prevention and control programs for IPIs in school‐age children.

## 2. Materials and Methods

### 2.1. Study Area and Period

The research was carried out at Mikara Primary School, which is located in the North Gondar Zone of the Amhara Regional State in Ethiopia. This area is located about 830 km northwest of Addis Ababa, the capital of Ethiopia, and approximately 103 km from the town of Gondar. Geographically, it lies at 13° 8 ^′^ N latitude and 37° 54 ^′^ E longitude, at an altitude of 2,850 meters above sea level. The district experiences an average annual rainfall between 1,900 and 2,400 mm, whereas the mean annual temperature ranges from 12°C to 20°C. According to the 2007 national census conducted by the Central Statistical Agency of Ethiopia, the North Gondar Zone has a total population of 905,680, consisting of 425,846 men and 479,834 women. Mikara Primary School, located in Debark, offers formal education from Grade 1 to Grade 8. At the time of the study, the school had a total student enrollment of 1,320.

### 2.2. Study Design

Between February and May 2024, a school‐based cross‐sectional epidemiological study was conducted to assess the prevalence of IPIs and to identify related risk factors among school‐aged children attending Mikara Primary School in northwest Ethiopia.

### 2.3. Study Population

The study population comprised school students who agreed to participate and gave their informed consent to collect stool samples, as well as to document their sociodemographic data and information on possible risk factors during the study period.

### 2.4. Inclusion and Exclusion Criteria

The study included schoolchildren who agreed to submit stool samples and provide information on relevant risk factors at the time of data collection. On the contrary, children who had taken anthelmintic or antiprotozoal drugs in the previous 2 weeks, as well as those who were seriously ill and therefore unable to provide sociodemographic details during data collection, were excluded from the study.

### 2.5. Variables

In this study, independent variables included sociodemographic characteristics, such as sex, age, grade level, and educational level and occupation of mothers, as well as possible risk factors, namely eating unwashed fruits and vegetables, availability of toilets, source of drinking water, handwashing habits before meals and after defecation, and shoe‐wearing practices. The dependent variable was the rate of IPIs among schoolchildren.

### 2.6. Sample Size Determination and Sampling Techniques

The sample size (*n*) was calculated using the single population proportion formula, n = Z^2^∗P (1 − P)/d^2^, where *P* denotes the prevalence of IPI reported in an earlier study [17], *d* is the acceptable sampling error margin, and *Z* is the standard normal value for a 95% confidence level (1.96). Substituting these parameters produced an initial sample size of 346. For the prevalence parameter, a value of 34.2% was taken from a study carried out at the University of Gondar Community School in northwest Ethiopia [18]. The sample size was calculated on the basis of a 95% confidence level and a 5% error margin. To accommodate possible nonresponse or nonadherence, an extra 5% was added to the initial estimate, yielding a final required sample of 363 participants. This total was then distributed across grade levels in proportion to the number of students enrolled in each grade. The study participants were ultimately chosen through systematic random sampling, using class rosters as the sampling frame.

### 2.7. Data Collection Methods

#### 2.7.1. Questionnaire Survey

A structured questionnaire was used to collect data from all study participants. To improve the reliability and validity of the information, the children were interviewed in their native language. The tool collected information on sociodemographic variables (such as sex, age, grade level, mother′s educational level and occupation), as well as potential risk factors for IPIs. The questionnaire was first prepared in English and then translated into Amharic, the local language, for administration during interviews. The participants′ responses were subsequently translated back into English for analysis, ensuring that the original concepts were preserved in both language versions.

#### 2.7.2. Stool Sample Collection

Fresh stool samples were obtained for parasitological evaluation. Before collection, children were instructed on hygienic stool collection methods and asked to provide approximately 3–4 g of newly passed stool in prelabeled collection containers, using the provided applicator sticks. For children aged 6–11 years, parents or guardians were involved to help with the collection. Each sample container was marked with a unique identification code to maintain accurate traceability. All samples were then transported to Debark General Hospital and examined using standard parasitological procedures.

##### 2.7.2.1. Direct‐Wet Mount Method.

Approximately 2 mg of each stool sample was mixed with a drop of physiological saline (0.85% NaCl). A drop of this suspension was then placed on a microscope slide, a few drops of iodine solution were added, and the preparation was covered with a coverslip. The slides were examined under a microscope following the World Health Organization (WHO) guidelines.

##### 2.7.2.2. Formol‐Ether Sedimentation Method.

One gram (1 g) of stool was introduced into a centrifuge tube containing 7 ml of 10% formalin. The sample was thoroughly mixed with an applicator stick until a uniform suspension was obtained. The suspension was then passed through a cotton gauze sieve into a beaker, after which the filtrate was poured back into the same centrifuge tube. Next, 3 ml of diethyl ether was added, the tube was securely capped, and the mixture was vigorously shaken to facilitate a thorough blending. The tube was centrifuged at 1500 rpm for 2 min. After centrifugation, the supernatant, which consists of layers of ether, particulate debris, and formalin, was carefully poured off and discarded, leaving the sediment at the bottom of the tube, which contained concentrated parasitic elements. This sediment was gently resuspended and then transferred onto a microscope slide using a Pasteur pipette. The slide was microscopically examined under 10× and 40× objectives by a laboratory technologist to identify any parasitic organisms [19].

### 2.8. Data Analysis

The information collected from the questionnaires and laboratory results was reviewed for completeness and internal consistency, then coded and entered into a computerized database. The data collected were analyzed using SPSS Version 23. First, descriptive statistics were generated, and the findings were presented as frequencies and percentages. Subsequently, a logistic regression analysis was applied to evaluate the strength of the association between potential risk factors and IPIs. An initial univariate logistic regression was performed to identify candidate variables, using a significance level of *p* ≤ 0.25 [16]. Variables that showed statistical significance in the univariate analysis were then included in a multivariate model to determine the independent predictors of IPIs among schoolchildren. A *p* value < 0.05, within a 95% confidence interval, was considered statistically significant.

## 3. Results

### 3.1. Sociodemographic Characteristics of the Study Participants

A total of 363 schoolchildren participated in this study, corresponding to a response rate of 100%. The ages of the participants ranged from 6 to 18 years, with a mean (± SD) of 11.4 ± 2.32 years. Among all participants, 156 (42.9%) were boys and 207 (57.1%) were girls. Regarding age categories, 166 (45.7%) were under 9 years of age, 152 (41.9%) were 9–12 years old, and 45 (12.4%) were 13 years or older. In terms of school grade, 202 (55.6%) attended Grades 1 to 4 and 161 (44.4%) were in Grades 5 to 8 (Table 1).

**Table 1:** Sociodemographic characteristics of the schoolchildren at Mikara Primary School from February to May 2024.

**Characterstics**	**Frequency**	**Percent**
Sex		
Male	156	42.9
Female	207	57.1
Age category		
Below 9 years	166	45.7
9–12 years	152	41.9
13 years and above	45	12.4
Grade level		
Grade 1–4	202	55.6
Grade 5–8	161	44.4
Mother occupation		
Howswife	113	31.1
Farmer	135	37.2
Merchant	61	16.8
government employee	54	14.9
Mothers educational status		
Illiterate	65	17.9
Primary education	163	44.9
Secondary education	90	24.8
College/university	45	12.4

### 3.2. Prevalence of IPIs Among Schoolchildren

Among the 363 students evaluated for IPIs, 147 (40.5%) tested positive for at least one type of intestinal parasite. Of these, intestinal protozoan infections accounted for a prevalence of 17.1% (62/363), whereas intestinal helminth infections had a prevalence of 23.4% (85/363). The prevalence of concurrent infection with two parasites in this study was 3.2%–3.3% (12/363). In total, seven different species of intestinal parasites were detected through direct‐wet mount microscopy and the formol‐ether concentration method. *Entamoeba histolytica/dispar* was the most common species identified, with a prevalence of 10.7% (39/363), followed by *Giardia lamblia* at 8.8% (32/363), *Ascaris lumbricoides* at 8.5% (31/363), hookworm species at 7.2% (26/363), *Trichuris trichiura* at 4.1% (15/363), Taenia species at 2.8% (10/363), and *Enterobius vermicularis* at 1.7% (6/363) (Table 2).

**Table 2:** Prevalence of intestinal parasitic species among schoolchildren in Mikara primary school from February to May 2024.

**Types of IPIs**	**Frequency** **n**	**Percent (%)**
Protozoa		
*E.histolytica*	39	10.7
*G.lamblia*	32	8.8
Helmenth		
*A.lumbricoides*	31	8.5
Hookworm	26	7.2
*T. trichuria*	15	4.1
Tania species	10	2.8
*E.vermicularis*	6	1.7
No. of parasites		
Double infection	13	3.3

### 3.3. Prevalence of IPIs in Relation to Sex and Grade Level of Students

**Figure 1:**
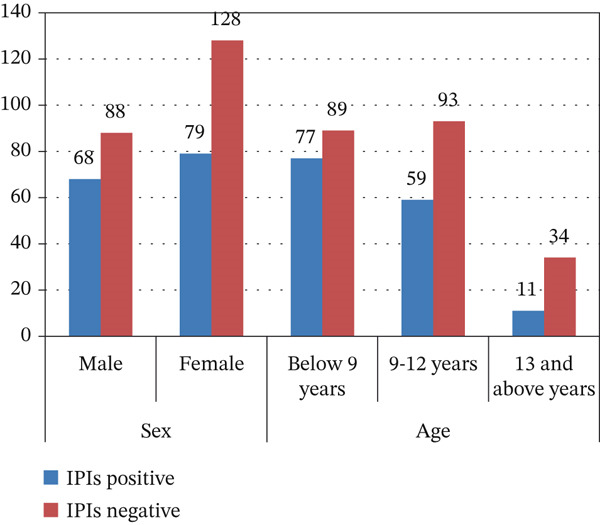
Prevalence of IPIs in relation to sex and age of students.

Figure 1 shows the distribution of IPIs among schoolchildren according to sex and grade level. When stratified by sex, male students exhibited a higher prevalence of IPI, 44.02% (68/156) than female students, 38.2% (79/207). In terms of age, the highest prevalence was observed in children younger than 9 years, 46.4% (77/166), whereas the lowest prevalence occurred among students aged 13 years and older, 24.4% (11/45).

### 3.4. Intestinal Parasites and Possible Risk Factors

Univariate analysis of sociodemographic variables and possible risk factors for IPIs showed that sex, age group, grade level, mother′s educational level, eating unwashed fruits and vegetables, type of sanitation facility, source of drinking water, hand washing before meals and after defecation, as well as raw meat consumption were significantly associated with IPIs (*p* < 0.25). However, in the multivariate analysis, only sex, age group, type of sanitation facility, source of drinking water and handwashing before meals remained independent predictors of IPIs (*p* < 0.05) (Table 3).

**Table 3:** Univariate and multivariate analysis of sociodemographic and risk factors associated with IPI among schoolchildren, Mikara Primary School (February 2024).

**Characterstics**	**No examined (%)**	**Positive (%)**	**COR (95% CI)**	**p**	**AOR (95% CI)**	**p**
Sex						
Male	156 (42.9)	68 (43.6)	4.3 (1.02–3.7)	0.018	2.02 (1.07–6.48)	0.042
Female	207 (57.1)	79 (38.2)	1		1	
Age category						
Below 9 years	166 (45.7)	77 (46.4)	5.23 (1.57–9.27)	~0.001	3.22 (1.29–7.25)	0.036
9–12 years	152 (41.9)	59 (38.8)	4.21 (1.36–7.79)		1.7 (0.76–5.46)	
13 years and above	45 (12.4)	11 (24.4)	1		1	
Grade level						
Grade 1–4	202 (55.6)	86 (42.5)	2.86 (1.36–4.82)	0.008	0.89 (0.36–2.72)	0.523
Grade 5–8	161 (44.4)	61 (37.9)	1		1	
Family occupation						
Howswife	113 (31.1)	49 (43.4)	1.89 (0.78–3.84)	0.838		
Farmer	135 (37.2)	73 (54.1)	0.48 (0.42–2.06)			
Merchant	61 (16.8)	22 (36.1)	0.56 (0.42–2.33)			
government employee	54 (14.9)	3 (5.56)	1			
Mother′s educational status						
Illiterate	65 (17.9)	39 (60)	3.8 (1.12–5.46)	~0.001	2.1 (0.74–4.25)	0.078
Primary education	163 (44.9)	81 (49.7)	2.7 (0.53–3.28)		1.3 (0.16–3.88)	
Secondary education	90 (24.8)	23 (25.6)	1.5 (0.31–2.79)		0.8 (0.18–2.87)	
College/university	45 (12.4)	4 (8.89)	1		1	
Eat unwashed fruits and vegetables.						
Always	198 (54.5)	86 (43.4)	2.8 (0.86–4.44)	0.008	1.01 (0.41–3.12)	0.321
Sometimes	142 (39.1)	56 (39.4)	1.52 (0.32–3.45)		0.51 (0.12–2.74)	
Never	23 (6.3)3	5 (21.7)	1		1	
The type of toilet used						
Open defecation	78 (21.5)	46 (58.9)	5.7 (1.78–7.89)	0.003	3.2 (1.52–5.84)	0.032
Public	66 (18.2)	38 (57.6)	2.4 (1.21–6.23)		1.8 (0.89–4.76)	
Private	219 (60.3)	63 (28.8)	1		1	
Source of drinking water						
River/spring	77 (21.2)	54 (70.1)	7.4 (3.85–8.89)	~0.001	4.2 (1.74–5.86)	0.023
Pipe	286 (78.8)	93 (32.5)	1		1	
Never washed.hands before having meal						
Never	83 (22.9)	52 (62.5)	5.6 (3.75–8.78)	~0.001	3.65 (1.56–7.25)	
Sometimes	182 (50.1)	75 (41.2)	3.6 (1.65–5.23)		1.36 (0.87–4.23)	0.011
Always	98 (26.9)	20 (20.4)	1		1	
Washing hands after defecation						
No	137 (37.7)	84 (61.3)	3.89 (1.32–3.76)	0.21	1.25 (0.14–2.57)	0.456
Yes	226 (62.3)	63 (27.8)	1		1	
Shoe‐wearing habit						
Sometimes	93 (25.6)	49 (52.7)	1.2 (0.45–2.14)	0.65		
Regularly	270 (74.4)	98 (36.3)	1			
Eating raw meat						
Yes	123 (33.9)	72 (58.5)	2.89 (1.21–3.56)	0.082	0.89 (0.45–2.76)	0.863
No	240 (66.1)	75 (31.3)	1		1	
Clean and cut nails						
Sometimes	149 (41.1)	82 (55)	2.1 (0.56–3.58)	0.562		
Always	214 (58.9)	65 (30.4)	1			

Male students had approximately 2.02 times higher odds of IPI than female students (AOR = 2.02, 95% CI 1.07–6.48). Similarly, schoolchildren younger than 9 years showed about 3.22 times higher odds of IPI (AOR = 3.22, 95% CI: 1.29–7.25) compared with those aged 13 years or older. Additionally, students who participated in open defecation experienced a 3.2‐fold increase in the odds of IPI (AOR = 3.20, 95% CI: 1.52–5.84) compared with those who used private latrines. In the same vein, students who drank water from rivers or streams had 4.2 times higher odds of infection (AOR = 4.20, 95% CI: 1.74–5.86) than those who used piped water. Furthermore, students who did not wash their hands before eating were 3.65 times more likely to have IPI (AOR = 3.65, 95% CI: 1.56–7.25) than those who reported washing their hands before meals (Table 3).

## 4. Discussion

This study examined how common IPIs are and which risk factors are associated with them among school‐aged children attending Mikara Primary School. The overall prevalence of IPI of 40.5% matched the 40.2% previously reported in the Gurage Zone of Ethiopia [20], however, this figure is still lower than the prevalence documented in Senbete and Bete towns, North Shoa, Ethiopia (52.3%) [17]; Adigrat Town, Northern Ethiopia (50.81%) [21]; Bahir Dar, Ethiopia (45%) [22]; and Rwanda (44.8%) [23]. On the contrary, this figure exceeded those reported in studies carried out in southern Ethiopia (27.1%) [24]; Delo‐Mena District in southeast Ethiopia (26.6%) [25]; East Arsi, Ethiopia (27.1%) [26]; Iran (21.5%) [27]; and Western Saudi Arabia (12%) [28].

The relatively high prevalence of IPIs observed in this study reflects a substantial burden of disease among school‐age children, despite the presence of numerous public health initiatives at both the regional (Amhara region) and local levels in Ethiopia. At the national level, multiple strategies have been introduced to improve population health, such as the Health Extension Program, which places trained health extension workers in communities to provide preventive and basic curative services at the household and community levels [29]. However, the persistently elevated prevalence of IPIs is likely driven by a combination of interconnected factors: incomplete geographical or population coverage of health services, limited uptake of available services by some households, inadequate compliance with recommended personal hygiene behaviors, insufficient sanitation facilities and unsafe water sources in schools and communities, and environmental conditions that favor ongoing transmission of intestinal parasites [30]. Furthermore, repeated contact with contaminated water and soil probably leads to frequent reinfections, weakening the long‐term effectiveness of deworming and other control strategies. Sociocultural norms and behaviors together with deficiencies in the scope, reach, or cultural relevance of health education, can further reduce the impact of current interventions, thus allowing IPIs to remain widespread among schoolchildren [31].

Another important finding in this study was the presence of simultaneous (dual) IPIs. The proportion of double infections (3.3%) was relatively lower than the 5.36% previously documented in the Mecha district of northwest Ethiopia [5], but exceeds the prevalence of 1.56% reported in a study conducted in northwest Ethiopia [32]. Discrepancies between studies may be attributable to differences in socioeconomic conditions, stool examination methodologies, sample size, and characteristics of the study population, as well as environmental sanitation levels [33].

The main IPIs detected in this study were *E. histolytica/dispar, G. lamblia, A.lumbricoides*, and hookworm. The prevalence of *E. histolytica/dispar* (10.7%) was similar to that found in a previous study in Adele town, East Arsi, Ethiopia (10.3%) [26], but lower than the rates reported in Gobgob, northwest Ethiopia (13%) [34]; Homesha District, Western Ethiopia (14.17%) [35]; and Bahir Dar, Ethiopia (24.5%) [36]. On the contrary, it was higher than the prevalences documented in Jawi, Ethiopia (5.9%) [37]. These variations are likely due to differences in drinking water quality and contamination, the consumption of raw or not washed vegetables, and variations in personal and environmental hygiene practices.

In the current study, the prevalence of *G. lamblia* (8.8%) was lower than the figures reported from Bahir Dar, Ethiopia (11.4%); Homesha District in northeast Ethiopia (12.65%) [35]; and Jawi Town, Ethiopia (19.95%) [37]. On the contrary, it was higher than the prevalence recorded in Delo‐Mena (2.0%) [26], Adigrat Town in Northern Ethiopia (2.29%) [22], and Birbir Town in Southern Ethiopia (4.8%) [25]. These differences can reflect variations in food hygiene practices, drinking water quality, and environmental sanitation between the respective study areas.

The prevalence of *A. lumbricoides* (8.5%) identified in the current study was similar to that reported in Birbir Town, southern Ethiopia (8.8%) [25], but lower than Sasiga District in southwest Ethiopia (22.7%) [37] and Adigrat Town in northern Ethiopia (19.1%) [22]. On the contrary, it exceeded the prevalence recorded in Homesha District, northwest Ethiopia (0.5%) [35] and in Jawi Town, Ethiopia (0.73%). These variations in prevalence can reflect differences in raw vegetable consumption, hand washing behavior after defecation and before eating, school waste disposal systems, drinking water sources, and general environmental sanitation.

The prevalence of hookworm infection identified in the current study (7.2%) was lower than that documented in Bahir Dar, Ethiopia (22.8%) [36]and 22 and in the Sasiga District, southeast Ethiopia (20.6%) [2], but higher than the rates reported in the Delo‐Mena District (0.8%) [38] and Glomekeda District, Northern Ethiopia (0.7%) [39]. These variations in hookworm prevalence between study areas can be explained by differences in the extent and effectiveness of mass drug administration, environmental sanitation, school‐based deworming programs, and student adherence to wearing shoes.

Several variables showed statistically significant associations with IPIs. Male students were infected more often than female students, consistent with findings from southwest and northern Ethiopia [2]. This pattern can be explained by the fact that boys spend more time outdoors, where they are more likely to encounter contaminated soil and water. Age was also identified as a key factor, aligning with Hawassa′s observations that younger children face higher risk, likely due to less advanced personal hygiene habits and behaviors that increase contact with infectious agents. Environmental and behavioral factors, such as open field solid waste disposal, the use of unsafe drinking water, and poor hand hygiene, were found to play a major role in the development of IPI, supporting the results of previous studies in different parts of Ethiopia and other countries [39]. In general, these results highlight the importance of improving environmental sanitation, ensuring access to microbiologically safe drinking water, strengthening school health education and addressing specific local behavioral and environmental risk factors to reduce the burden of IPIs among school‐aged children.

## 5. Conclusions

The results of this study revealed a substantial burden of IPIs among the students at Mikara Primary School. Helminth infections (23.4%) occurred more frequently than protozoan infections (17.1%). Seven species of intestinal parasites were detected: *E. histolytica/disar, G. lamblia, A. lumbricoides,* hookworm, *Trichuris trichiura*, Taenia species, and *Strongyloides stercoralis*. Of these, *E. histolytica/disar, G. lamblia, A. lumbricoides*, and hookworm were the most commonly encountered IPI in this population. The investigation also examined sociodemographic and behavioral factors linked to IPI. Sex, age category, type of sanitation facility, drinking water source, and handwashing before meals were identified as significant predictors of IPI among children. Collectively, these findings highlight the need for stakeholders to develop and apply evidence‐based control and prevention measures for IPIs in the area, as well as to strengthen health education and awareness programs for students and their parents.

## Author Contributions

G.A.A. conceived and designed the study, extracted and analyzed the data, interpreted the findings, and drafted the manuscript. Y.A. contributed to the selection, quality assessment, and acquisition of the study.

## Funding

No funding was received for this manuscript.

## Ethics Statement

Ethical approval for this study was obtained from the Debark University Research Ethics Committee (Ref. No: REC/DKU/041/2024). Additional permission to conduct the study was granted by the Mikara Primary School administration. Written informed consent was obtained from the parents or legal guardians of all schoolchildren who participated in the study at Mikara Primary School, as all participants were under 16 years of age. In addition, verbal assent was obtained from the children after explaining the purpose, procedures, benefits, and potential risks of the study in an age‐appropriate manner. Participation was entirely voluntary, and children were free to withdraw from the study at any time without any consequences. Confidentiality of all participants′ information was strictly maintained throughout the study.

## Conflicts of Interest

The authors declare no conflicts of interest.

## Data Availability

The data sets used and/or analyzed during the current study are available from the corresponding author on reasonable request.
